# Ultrasound Stimulation Modulates Voltage-Gated Potassium Currents Associated With Action Potential Shape in Hippocampal CA1 Pyramidal Neurons

**DOI:** 10.3389/fphar.2019.00544

**Published:** 2019-05-22

**Authors:** Zhengrong Lin, Xiaowei Huang, Wei Zhou, Wenjun Zhang, Yingzhe Liu, Tianyuan Bian, Lili Niu, Long Meng, Yanwu Guo

**Affiliations:** ^1^Paul C. Lauterbur Research Center for Biomedical Imaging, Institute of Biomedical and Health Engineering – Shenzhen Institutes of Advanced Technology, Chinese Academy of Sciences, Shenzhen, China; ^2^Key Laboratory of E&M, Ministry of Education and Zhejiang Province, Zhejiang University of Technology, Hangzhou, China; ^3^Sino-Dutch Biomedical and Information Engineering School, Northeastern University, Shenyang, China; ^4^The National Key Clinic Specialty, The Engineering Technology Research Center of Education Ministry of China, Guangdong Provincial Key Laboratory on Brain Function Repair and Regeneration, Department of Neurosurgery, Zhujiang Hospital, Southern Medical University, Guangzhou, China

**Keywords:** ultrasound stimulation, potassium currents, action potential, hippocampal neurons, neuro-modulation

## Abstract

Potassium channels (K^+^) play an important role in the regulation of cellular signaling. Dysfunction of potassium channels is associated with several severe ion channels diseases, such as long QT syndrome, episodic ataxia and epilepsy. Ultrasound stimulation has proven to be an effective non-invasive tool for the modulation of ion channels and neural activity. In this study, we demonstrate that ultrasound stimulation enables to modulate the potassium currents and has an impact on the shape modulation of action potentials (AP) in the hippocampal pyramidal neurons using whole-cell patch-clamp recordings *in vitro*. The results show that outward potassium currents in neurons increase significantly, approximately 13%, in response to 30 s ultrasound stimulation. Simultaneously, the increasing outward potassium currents directly decrease the resting membrane potential (RMP) from −64.67 ± 1.10 mV to −67.51 ± 1.35 mV. Moreover, the threshold current and AP fall rate increase while the reduction of AP half-width and after-hyperpolarization peak time is detected. During ultrasound stimulation, reduction of the membrane input resistance of pyramidal neurons can be found and shorter membrane time constant is achieved. Additionally, we verify that the regulation of potassium currents and shape of action potential is mainly due to the mechanical effects induced by ultrasound. Therefore, ultrasound stimulation may offer an alternative tool to treat some ion channels diseases related to potassium channels.

## Introduction

Potassium channels (K^+^), a high degree of diversity and ubiquity embedded within cellular membranes of neurons, are one of the most fundamental and important component in the function of ion channels (Mackinnon, 2003; Mckeown et al., 2008; Coetzee et al., 2010). Potassium channels-mediated trans-membrane ion currents play a critical role in neuronal resting membrane potential (RMP) and action potential repolarization (Schwindt et al., 1988; Kolb, 1990; Pongs, 1999; Vacher et al., 2008; Johnston et al., 2010). Dysfunction of K^+^ channels has been found to associate with many diseases, such as long QT syndrome, episodic ataxia and epilepsy (Adelman et al., 1995; Shieh et al., 2000; Giudicessi and Ackerman, 2012; Maljevic and Lerche, 2014). A common feature of these diseases is associated to a reduction of potassium currents or loss of membrane potential repolarization (Lawson, 2000a; Sandhiya and Dkhar, 2009). Functional modulation of potassium channels is of great significance for clinical treatment of these relevant diseases (Lawson, 2000b; Maljevic and Lerche, 2013).

Ultrasound stimulation has emerged as a promising approach to modulate the nervous system that is capable of transmitting through the intact skull non-invasively and focusing acoustic energy in the deep brain nuclei precisely (Tufail et al., 2010, 2011; Bystritsky and Korb, 2015; Leinenga et al., 2016). It has been proven that neural activity could be evoked by ultrasound stimulation from model organism to human beings (Tufail et al., 2010; Legon et al., 2014; Li et al., 2016; Zhou et al., 2017; Lin et al., 2018). More importantly, various studies have demonstrated that regulation of neuronal activity induced by ultrasound stimulation resulted from activating ion channels with different types (Bystritsky et al., 2011). Tyler et al. (2008) illustrated that ultrasound could induce electrical activity in neurons by activating voltage-gated sodium channels and voltage-gated calcium channels. Chapman et al. (1980) demonstrated that ultrasound stimulation could modulate rates of influx and efflux of potassium ions in rat thymocytes *in vitro*. Kubanek et al. (2016) showed that ultrasound stimulation could directly modulate the trans-membrane currents flowing through the mechano-sensitive ion channels (sodium channels and potassium channels) expressed in *Xenopus oocytes*. Rencently, we reported that ultrasound stimulation enables to increase neuronal excitability by increasing the total sodium currents and modulating the sodium channels kinetics (Lin et al., 2018).

Although growing interest and research in ultrasound-induced modulation of different ion channels, little is known about the effect of ultrasound stimulation on potassium channels of neurons. In this paper, we first investigate the modulation of ultrasound stimulation on potassium channels in hippocampal pyramidal neurons using *in vitro* patch-clamp recording in brain slices. In neurons, action potentials play a central role in cell-to-cell communication and finely tuned by the diverse populations of ion channels expressed in cellular membranes which act as a molecular switch for controlling the activity of targeted neurons (Llinás, 1988; Hille, 2001; Bean, 2007). Small change in different types of ion channels conductance can lead to dramatic changes in action potential shape and even overall excitability (Swensen and Bean, 2005; Amendola et al., 2012; Goldman et al., 2013). We further quantify how the increased potassium currents induced by ultrasound stimulation impact AP properties and neural excitability. Moreover, the underlying mechanisms remains unknown and it is necessary to further investigate the candidate mechanisms including thermal effect, mechanical effect and cavitation effect on single neuron.

## Materials and Methods

### Ultrasound Application

**FIGURE 1:**
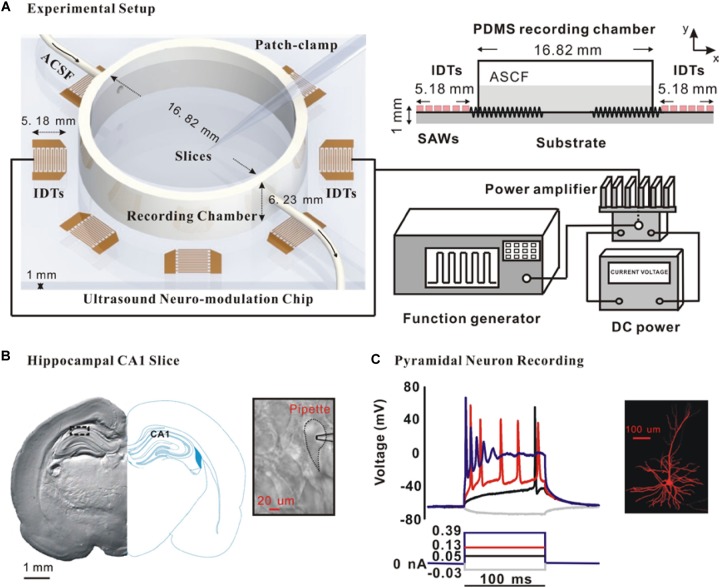
Schematic of the experimental system and the whole-cell recording of pyramidal neurons in the hippocampal CA1 slices. **(A)** Schematic of the entire experimental system and a cross-sectional diagram of the exposure setting with the actual dimensions. **(B)** Photomicrograph of a coronal section of hippocampal CA1 slice and a typical pyramidal neuron for the whole-cell recording. **(C)** Action potential in response to prolonged depolarizing current injection is one important feature to identify pyramidal neuron. Moreover, intracellular injection with 0.25% biocytin shows the morphology of a typical CA1 pyramidal neuron.

In this study, we developed an ultrasound neuro-modulation chip by the standard MEMS (Microelectromechanical systems) processes. This chip was a kind of surface acoustic wave device as described before (Lin et al., 2018) and generate a uniform acoustic field with a clear acoustic boundary which can precisely modulate neuronal activity in the region-specific slices. Figure 1A shows the ultrasound neuro-modulation chip which was compatible with the patch-clamp recording system. The response of the hippocampal CA1 slices to the ultrasound stimulation also could be recorded in a real time manner. Briefly, a pair of interdigital transducers (IDTs) at the resonant frequency of 27.38 MHz was excited to generate standing surface acoustic waves (SSAWs) to stimulate the neurons. The acoustic energy was localized to the substrate surface, facilitating the stimulation of the neurons located at a polydimethylsiloxane ring-shaped (PDMS, Sylgard 184, Dow Corning, United States) recording chamber with a relatively small input power (Klemic et al., 2002). Continuous radio frequency (RF) signals were generated by an arbitrary waveform generator (AFG 3102, Tektronix, Beaverton, OR, United States), amplified by a power amplifier (ZHL-1-2W+, Mini-Circuits, Brooklyn, NY, United States) and then applied to both IDTs. The spatial-peak-pulse-average intensity (I_SPPA_) generated by IDTs in the experiments was approximately 465 mW/cm^2^, measured by a Laser Doppler Velocimetry (UHF-120 Ultra High-Frequency Vibrometer, Polytec, Germany).

### Slices Preparation

All animal experiments were performed according to the guidelines approved by the Use Committee and the Ethics Committee of Shenzhen Institutes of Advanced Technology, Chinese Academy of Sciences. Postnatal day 13–15 (P13–15) Sprague-Dawley (SD) rats were used in this study. Animals were sacrificed using a rodent guillotine under deep anesthesia with 20% urethane (10 ml/kg). The brain was rapidly removed from the skull and immersed in ice-cold, oxygenated high-sucrose cutting solution (0–2°C) containing (in mM): 60 NaCl, 3 KCl, 7 MgCl_2_, 1.25 NaH_2_PO_4_, 25 NaHCO_3_, 10 D-glucose, 115 sucrose, and 0.5 CaCl_2_. Coronal slices (300 μm thick) containing the hippocampus CA1 area were prepared with a Vibratome (VT-1200 Series, Leica). The hippocampal slices were equilibrated and incubated in the artificial cerebrospinal fluid (ACSF) containing (in mM): 126 NaCl, 2.5 KCl, 1 MgCl_2_, 1.25 NaH_2_PO_4_, 26 NaHCO_3_, 10 D-glucose, 2 sodium pyruvate, 0.5 L-Ascorbic acid, and 2 CaCl_2_, saturated continuously with 95% O_2_-5% CO_2_ and maintaining the temperate at 35°C. The osmolality of ACSF was 300–310 Osm/kg.

### Electrophysiological Recording

After incubation, brain slices were transferred to the recording chamber. The slices were per-fused with ACSF flowing at a rate of 2–3 ml/min which was maintained at 30°C by an automatic temperature controller (TC-344B, WARNER) in the experiments. As shown in Figure 1B, the CA1 pyramidal neurons in the hippocampus were visualized by the morphology using an infrared DIC microscope (FN-S2N, Nikon, Japan). Further identification was carried out by the intra-cellular injection of 0.25% biocytin (Figure 1C).

Traditional whole-cell voltage-clamp and current-clamp recording were performed to record the isolated K^+^ currents and evoked action potentials of pyramidal neurons using a patch-clamp amplifier EPC 10 (HEKA, Germany). In whole-cell recording, series resistance (Rs) compensation was used to correct membrane voltage errors under conditions of high access resistance between pipette and cell interior. 70–90% compensation was applied to stabilize the final Rs value to 1–3 MOhm. Membrane potentials were corrected for junction potentials by applying the appropriate offset potential before recording. Leakage and capacitive currents were subtracted on-line. Patch glass microelectrodes were pulled by a micropipette puller (P-97, Sutter Instrument Co., Novato, CA, United States) and the resistance ranged from 5 to 10 MΩ after filling with the internal solution. To measure the isolated outward potassium currents in voltage-clamp mode, neurons were held at −70 mV, current-voltage (I-V) curve was obtained by applying depolarizing from −60 to +60 mV for 200 ms in 10 mV increment (Schwindt et al., 1988; Kolb, 1990; Pongs, 1999; Vacher et al., 2008; Johnston et al., 2010). The experiments were performed at room temperate (22–25°C). TTX (1 μM) and CdCl_2_ (0.3 mM) were added to block sodium channels, Ca^2+^ current and Ca^2+^-dependent K^+^ currents. Furthermore, recording currents were further identified by a pharmacological blocker, and the recording currents could be fully blocked by the application of 1 mM 4-AP and 30 mM TEA-Cl. The outward potassium currents recording internal solution contained the following (in mM): 120 KCl, 1 MgCl_2_-6H_2_O, 1 CaCl_2_, 10 HEPE, 10 EGTA, 2 Mg-ATP, pH 7.2 adjusted with KOH and HCl, the osmolality was 300 Osm/kg. To investigate the influence of ultrasound stimulation on membrane properties of individual pyramidal neuron, action potentials were elicited by different injected current pulses (ranging between −100 to 400 pA, 100 ms timescale, 10 pA increment and 0.2 Hz frequency) using a whole-cell current clamp. Active membrane properties and the passive intrinsic properties were characterized as a function of neuronal activity (Franzen et al., 2015; Hong et al., 2016). F-I slope was defined as the slope of relation curve between the firing frequency and injected currents). Threshold current, also called rheobase, was defined as the minimum amount of current required for neurons to generate an action potential more than 50% of the time across 20 repetitive. RMP was defined as the actual measured value without considering the pipette offset potential. The current-clamp internal solution contained the following (in mM): 140 K-gluconate, 4.5 MgCl2, 5 EGTA, 4 Mg-ATP, 0.3 GTP, 4.4 phosphocreatine disodium salt hydrate, and 9 HEPEs.

### Statistical Analysis

All electrophysiological data were analyzed offline using Patchmaster acquired software (HEKA, Germany), Clampfit analysis software (version 10.3; Molecular Devices, Silicon Valley, CA, United States); MiniAnalysis, MATLAB (version R2014b; The Math Works, Natick, MA, United States) and Origin 8.0. All numerical results are presented as mean ± standard error of the mean (SEM). Student’s paired *t*-test and one-way ANOVA were used for all statistical analyses using the statistics software, SPSS ver.13.0. Statistical significance was defined as a value of *p* < 0.05.

## Results

### Ultrasound Stimulation Directly Increased the Outward Potassium Currents of CA1 Pyramidal Neurons

**FIGURE 2:**
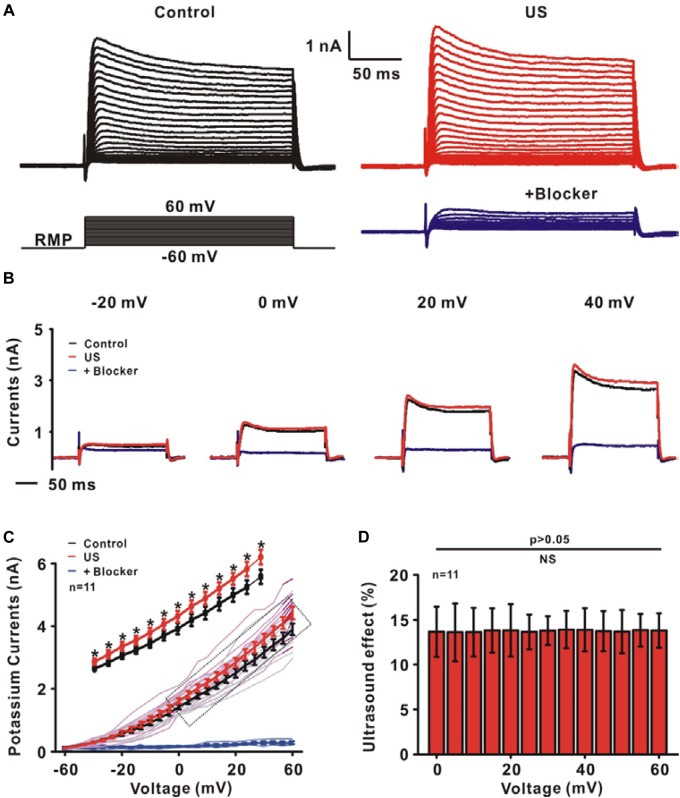
The effect of ultrasound stimulation on voltage-gated potassium currents of pyramidal neurons in CA1 hippocampus slices. **(A)** Representative outward potassium currents recorded in the control (black) and ultrasound stimulation (red). The currents were elicited by a protocol in which cells were depolarized to a variety of potentials (–60 to +60 mV) from a holding potential of –70 mV and the currents could be blocked by 30 mM TEA-Cl and 1 mM 4-AP (blue). **(B)** The individual current traces correspond to the currents measured at voltage steps of –20, 0, 20, and 40 mV in control (black), US (red) and blocker (blue) group. **(C)** I–V curves of outward potassium currents showed that ultrasound stimulation increased the outward potassium currents (^∗^*p* < 0.05). **(D)** The same relative changes of the outward potassium currents at different voltage steps were observed.

To test whether ultrasound stimulation could modulate ion channels and neuronal activity in rat slices, an ultrasound stimulation system consisting of an ultrasound neuro-modulation chip and patch-clamp recording system was established to stimulate hippocampal CA1 slices *in vitro* and to record the electrophysiological activity of neurons synchronously, as shown in Figure 1A. The pyramidal neurons, located at the stratum pyramidal of hippocampus (Figure 1B), were identified by both firing pattern in response to current injections and typical morphology (Figure 1C). The ultrasound-induced modulatory response of ion channels was recorded using whole-cell voltage-clamp. Compared to the control group, ultrasound stimulation for 30 s duration on pyramidal neurons caused a significant increment in the peak amplitude of outward potassium currents, approximately 13% (Figure 2A–C). Moreover, Figure 2D shows that the relative change of outward potassium currents induced by ultrasound stimulation at different voltage steps did not show significant difference, indicating that the increment of potassium currents was caused by ultrasound stimulation, rather than electronic stimulation by holding voltage. Therefore, the results demonstrated that ultrasound stimulation was capable of modulating the potassium channels directly.

### Ultrasound Modulated the Evoked Firing in CA1 Pyramidal Neurons

**FIGURE 3:**
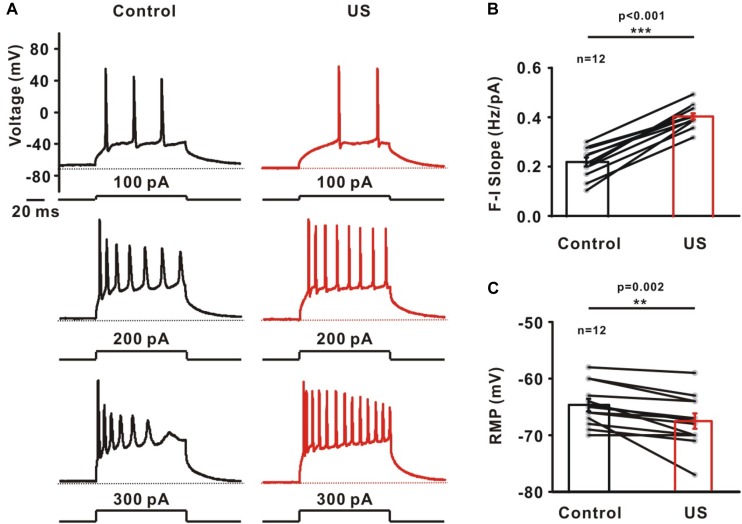
The effect of ultrasound stimulation on evoked firing of pyramidal neurons in CA1 hippocampus slices. **(A)** Representative voltage traces of evoked action potentials in response to current injections (100, 200, and 300 pA) in control (balck) and ultrasound stimulation (red). Dashed lines located at resting membrane potential (RMP) of neurons. **(B)** Collected data on the slope of firing frequency-currents relationship (F-I slope) showed that ultrasound stimulation significantly increased the firing frequency of pyramidal neurons (^∗∗∗^*p* < 0.001). Slope was determined by linear fit from 0 to 300 pA. **(C)** The effect of ultrasound stimulation on RMP was significantly decreased (^∗∗^*p* < 0.01).

Potassium channels play a prominent role in determining intrinsic neuronal excitability, firing threshold and RMP, etc. Further experiments were carried out to investigate the effects of increased potassium currents induced by ultrasound stimulation on firing properties using whole-cell current-clamp recording (Figure 3A). The action potentials in pyramidal neurons were evoked by different current injections. Figure 3B shows the slope of the relationship between frequency and injected currents (F-I slope). The results indicated that ultrasound stimulation leads to the increased firing frequency of neurons (Control: 0.22 ± 0.02 Hz/pA; US: 0.41 ± 0.01 pA, ^∗∗∗^*p* < 0.001, *n* = 12). In collected data, the RMP also decreased from −64.67 ± 1.10 mV to −67.51 ± 1.35 mV after ultrasound stimulation (^∗∗^*p* < 0.01, Figure 3C, *n* = 12).

### Regulation of AP Properties Associated With Potassium Channels by Ultrasound in CA1 Pyramidal Neurons

**FIGURE 4:**
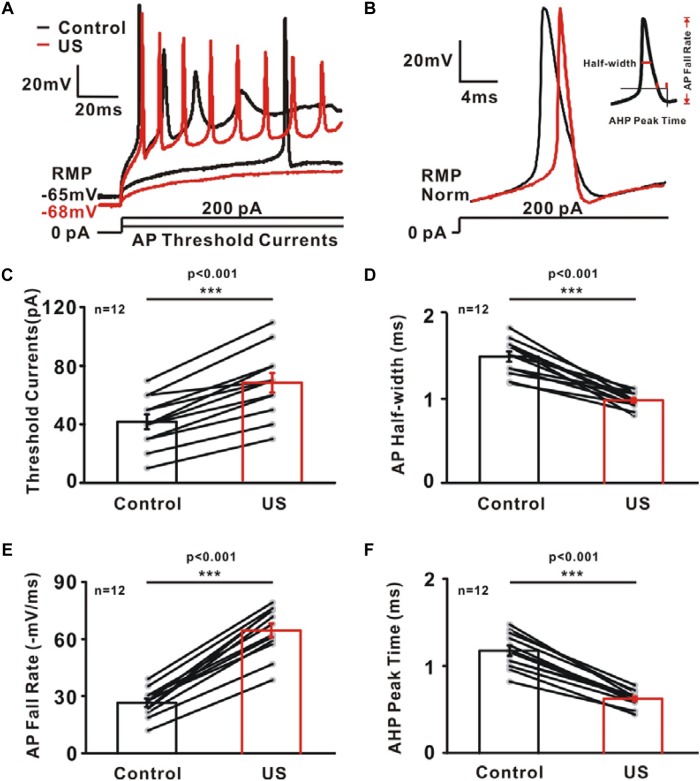
The effect of ultrasound stimulation on action potential (AP) properties of pyramidal neurons in CA1 hippocampus slices. **(A)** Superimposed voltage traces in response to sustained threshold current (50 pA) and above threshold current (200 pA). Threshold current is defined as the minimum amount of current injection required for neuron to generate action potential. In contrast to the voltage traces of control (black), ultrasound stimulation (red) failed to generate action potential in the threshold current injection and generate more action potential in 200 Pa current injection. **(B)** Representative voltage trace of single AP in control (black) and US (red) group evoked by sustained above threshold current injections (200 pA). AP properties were further calculated and plotted as a function of potassium currents. **(C–F)** A population of 12 neurons showing the increment of threshold current **(C)** and AP fall rate **(E)** while the reduction of AP half-width **(D)** and AHP peak time **(F)** by ultrasound stimulation (^∗∗∗^*p* < 0.001, paired *t*-test).

The increased potassium currents observed in pyramidal neurons may also alter the shape of single action potential. To test this hypothesis, further experiments were carried to investigate AP properties consisting of threshold current, AP half-width, AP fall rate and AHP peak time (Figure 4A,B). Ultrasound stimulation caused a significantly increment in the threshold current (Control: 41.67 ± 5.05 pA; US: 68.33 ± 6.61 pA, ^∗∗∗^*p* < 0.001, Figure 4C, *n* = 12), AP fall rate (Control: 26.44 ± 2.1 mV/ms; US: 64.21 ± 3.63 mV/ms, ^∗∗∗^*p* < 0.001, Figure 4E, *n* = 12) as well as a reduction in the AP half-width (Control: 1.49 ± 0.06 ms; US: 0.97 ± 0.03 ms, ^∗∗∗^*p* < 0.001, Figure 4D, *n* = 12) and AHP peak time (Control: 1.17 ± 0.06 ms; US: 0.62 ± 0.03 ms, ^∗∗∗^*p* < 0.001, Figure 4F, *n* = 12) of neurons in hippocampal CA1 slices.

### Regulation of Passive Properties by Ultrasound Stimulation in CA1 Pyramidal Neurons

**FIGURE 5:**
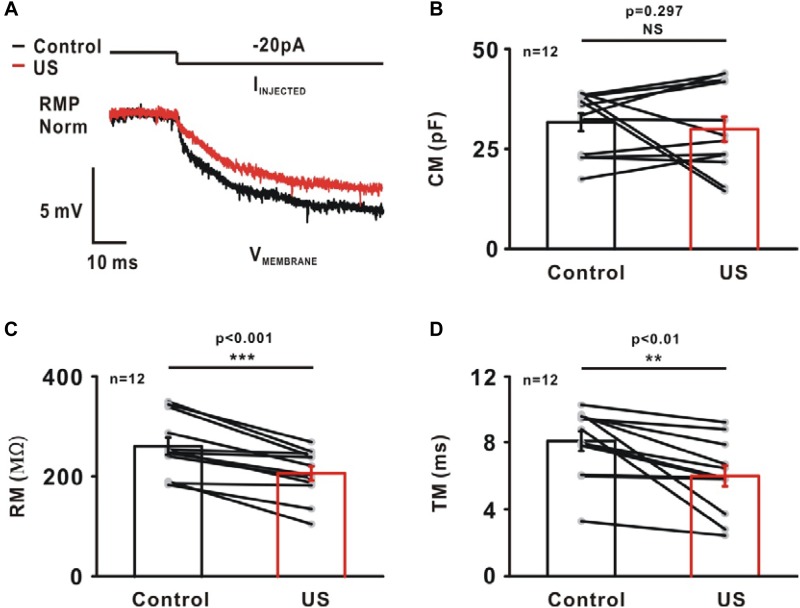
The effect of ultrasound stimulation on passive properties of pyramidal neurons in CA1 hippocampus slices. **(A)** An example of representative voltage trace of pyramidal neuron in response to a small hyperpolarizing current (−20 pA). A single exponential was fitted to a 10 ms time window of voltage trace to calculate passive membrane properties, membrane capacitance (CM), membrane input-resistance (RM) and membrane time constant (TM). Ultrasound stimulation had little effect on CM (**B**, Control: 31.58 ± 2.22 pF; US: 29.82 ± 3.13 pF, *p* = 0.297, paired *t*-test). However, ultrasound stimulation caused a reduction in RM (**C**, Control: 259.75 ± 17.24 MΩ; US: 205.89 ± 14.11 MΩ, ^∗∗∗^*p* < 0.001, paired *t*-test) and TM (**D**, Control: 8.07 ± 0.59 ms; US: 5.98 ± 0.62 ms, ^∗∗^*p* < 0.01, paired *t*-test).

The passive intrinsic properties, including the membrane capacitance (CM), membrane input resistance (RM) and membrane time constant (TM), were highly relevant to the generation of APs. These properties were characterized by injecting a small hyperpolarizing current into the soma of neurons (−20 pA, Figure 5A). The results shows there was no significant change in CM (Control: 31.58 ± 2.22 pF; US: 29.82 ± 3.13 pF, NS *p* = 0.297, Figure 5B, *n* = 12). However, compared to the control, the RM (Control: 259.75 ± 17.24 MΩ; US: 205.89 ± 14.11 MΩ, ^∗∗∗^*p* < 0.001, Figure 5C, *n* = 12) and the TM (Control: 8.07 ± 0.59 ms; US: 5.98 ± 0.62 ms, ^∗∗^*p* < 0.01, Figure 5D, *n* = 12) decreased significantly in the presence of ultrasound.

### Underlying Mechanisms of Ultrasound Stimulation in CA1 Pyramidal Neurons

**FIGURE 6:**
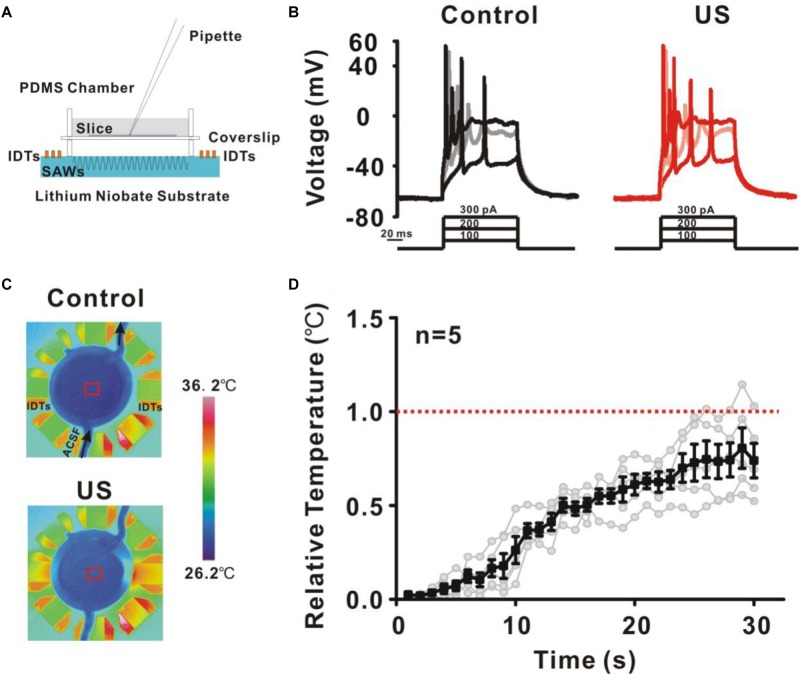
The underlying mechanisms of ultrasound stimulation on modulation of potassium channels in pyramidal neurons. **(A)** A new recording chamber was developed to isolate ultrasound waves which consisting of a cavity and sputtered to a lithium niobate substrate. **(B)** Representative voltage traces recorded from pyramidal neurons in response to a sequence of sustained currents injection (100, 200, and 300 pA). The result showed that the isolated ultrasound has no effect on the activity of neurons. **(C)** Temperature elevation was monitored during ultrasound stimulation in recording chamber using a thermal infrared imager. **(D)** The temperature profile showed that the temperature elevation was relatively small (less than 0.74 ± 0.09°C) during 30 s ultrasound stimulation.

Ultrasound has been reported to have a wide range of physical effects on the nervous system, such as thermal effect, mechanical effect or cavitation effect. The underlying mechanisms of ultrasound stimulation on the modulation of ion channels and neural activity remain unknown. Further experiments were carried out to investigate the biophysical effects of ultrasound. Substrate vibration-induced piezoelectric effects of the ultrasound neuro-modulation chip may have an effects on the potassium currents and neuronal activity (Yuchao et al., 2013). Air chamber was established to eliminate the ultrasound stimulation (Figure 6A) since ultrasound wave at high frequency could not propagate through the air medium. The result shows that no change in neural activity was detected, indicating the modulation of neurons was directly dependent on ultrasound stimulation, rather than electrical stimulation induced by the piezoelectric effects (Figure 6B). Thermal effect induced by ultrasound stimulation was another candidate responded for neuro-modulation. The experiment was further carried out to monitor the temperature elevation during the process of ultrasound stimulation (Figure 6C). As shown in the Figure 6D, small temperature elevation (less than 0.74 ± 0.09°C) was detected. Additionally, acoustic cavitation was highly dependent on the acoustic intensity and acoustic frequency. For 27.38 MHz, it is difficult to generate acoustic cavitation at such a high frequency in the absence of microbubbles, especially for such a small input power. These results indicated that the mechanical effects induced by ultrasound were the main reason for the modulation of potassium currents and the shape of action potential.

## Discussion

This study demonstrated the feasibility of ultrasound neuro-modulation on outward potassium currents and the regulation of AP properties in pyramidal neurons of hippocampal slices. The results show that ultrasound stimulation could directly increase outward potassium current, approximately 13%. Further data indicates that ultrasound enables to decrease the RMP as well as regulate AP properties, leading to the increment of threshold current, AP fall rate and the reduction of AP half-width, AHP peak time. Moreover, the regulation of potassium currents and the shape of action potential were mainly induced by the mechanical effect of ultrasound.

The ultrasound wave in the experiments is generated by an ultrasound neuro-modulation chip made of IDTs on a LiNbO_3_ substrate using a standard MEMS technology (Sritharan et al., 2006; Friend and Yeo, 2011; Meng et al., 2011). The ultrasound generated by IDTs has a clear acoustic boundary and the neurons in the region-specific slices can be stimulated precisely. Compared to the clinical ultrasound, the frequency used in the experiment (27.38 MHz) is relatively high. It is an anticipated that neural activity is highly dependent on the acoustic intensity (acoustic pressure, pulse duration and pulse repetition frequency) (Tufail et al., 2010; Menz et al., 2013; Li et al., 2016). Furthermore, we fabricated the similar neuro-modulation chip with a lower resonant frequency of 8.7 MHz to investigate the influence of the acoustic frequency on the potassium currents and neural activity. The results demonstrate that the ultrasound at 8.7 MHz also enable to increase the potassium currents and modulate the action potential properties in hippocampal CA1 pyramidal neurons using the same parameters. Thus, the similar results were acquired indicating that the ultrasound frequency has little influence on neuronal activity and potassium channels. Furthermore, the energy of surface acoustic waves is confined to the substrate surface, facilitating the stimulation of the neurons with a relatively small input power. As ultrasound neuro-modulation chip is compatible with patch clamp and standard calcium imaging, this chip is readily to study the effects of ultrasound on modulation of neuronal activity and ion channels.

As a mechanical pressure waves, the interaction between ultrasound and neurons is relatively complex. Ultrasound induced thermal effect, mechanical effect and cavitation is considered to be related to the neuronal activity (Bystritsky et al., 2011; Tyler, 2011; Plaksin et al., 2014). Previous studies have demonstrated that the small temperature elevation could not affect the neuronal activity in brain slice (Rinaldi et al., 1991; Bachtold et al., 1998; Tufail et al., 2010; Yoo et al., 2011). Our previous experiment based on the *C. elegans* shows that the AFD neurons, a thermal sensitive neurons, cannot be activated by the ultrasound stimulation, indicating that the response of the worms was not due to the temperature elevation (Zhou et al., 2017). We also stimulated the neuron in hippocampal slice using the heated ACSF (5°C temperature elevation) by water bath. The result shows 5°C elevation of ACSF perfusion has no significant effect on the spontaneous activity and could not evoke the firing of recording neurons, indicating that a small temperature elevation could not activate the neurons (Zhou et al., 2017). Furthermore, we carried out the experiment to monitor the temperature change during ultrasound stimulation using a thermal infrared imager. The result shows that ultrasound stimulation caused a relatively small temperature elevation in the recording chamber with ultrasound stimulation for 30 s (less than 0.74 ± 0.09°C, Figure 6D). Additionally, acoustic cavitation may be another reason for the ultrasound-induced modulation of neuronal activity (Wall et al., 1951; Menz et al., 2013). It was difficult to generate acoustic cavitation at 27.38 MHz with a small acoustic intensity, in the absence of microbubbles. Therefore, the mechanical effect of ultrasound was primary reason leading to the modulation of potassium currents and shape of action potential in hippocampus neurons.

The results demonstrated that ultrasound-induced mechanical effect was capable of modulating the potassium channels and regulating the AP properties of neurons directly. In mammalian neurons, there is a particularly large diversity of mechano-sensitive channels (Kuang et al., 2015). Numerous potassium channels (i.e., Shaker (Kv1.1); Ca^2+^-activated K^+^; TREK1; TRAAK; HCN2) have been found to be sensitive to mechanical stimulus (Maingret et al., 1999; Lin et al., 2007; Zhao and Sokabe, 2008; Brohawn et al., 2012; Hao et al., 2013; Gu and Gu, 2014). Recent studies have indicated that ultrasound stimulation can modulate the *trans*-membrane currents flowing TREK-1, TREK-2, TRAAK channels expressed in *Xenopus oocytes* (Kubanek et al., 2016). Ultrasound could induce behavioral responses by activation mechano-sensitive TRP-4 channel, a ion channel required for touch sensation in *C. elegans* (Ibsen et al., 2015; Kubanek et al., 2018). Our previous works indicated that the cultured neurons expressed with MscL, a mechano-sensitive channel from *Escherichia coli*, could be activated by low-pressure ultrasound (Ye et al., 2018). Transcriptional activities induced by ultrasound also have been investigated in mechano-sensitive Piezo1 channel expressed mammalian cells (Pan et al., 2018). Therefore, the underlying mechanism of ultrasound neuromodulation on neuronal activity may contribute to the activation of ion channels, especially mechano-sensitive channels.

Continuous ultrasound waves generated by ultrasound neuro-modulation chip were used in the experiments. The previous studies have shown that the modulatory effects were bimodal, whereby the neuronal activity could either be activated or suppressed, which was highly dependent on the acoustic parameters, such as acoustic pressure, pulse duration, pulse repetition frequency etc. (Pan et al., 2018). Further experiments are needed to be carried out to investigate the relationship between the modulatory effect of ion channels and acoustic parameters. The acoustic parameter also could be further optimized to modulate the neural activity more efficiently. By modulation of the potassium currents using ultrasound, some diseases related to potassium ion channels, such as long QT syndrome, epilepsy might be treated and ultrasound stimulation might pave a new way for clinical applications.

## Conclusion

Using whole-cell patch-clamp recording *in vitro*, the results indicate that it is feasible to modulate potassium channels by ultrasound stimulation in hippocampal slices. During the ultrasound stimulation, the increment of outward potassium currents and a regulation of action potential shape could be achieved. Moreover, the opening of potassium channels is responded to the mechanical effect induced by ultrasound. These observations provide the support for its effectiveness of ultrasound neuro-modulation on ion channels and may offer an alternative tool to treat some diseases related to potassium channels.

## Ethics Statement

All animal experiments were performed according to the guidelines approved by the Use Committee and the Ethics Committee of Shenzhen Institutes of Advanced Technology, Chinese Academy of Sciences.

## Author Contributions

ZL, XH, LN, and LM designed the experiments. ZL, XH, WZhou, and WZhang conducted the experiments. ZL, WZhou, YL, and TB managed the experimental animals and laboratory. ZL, LN, LM, and YG wrote the manuscript and revised manuscript.

## Conflict of Interest Statement

The authors declare that the research was conducted in the absence of any commercial or financial relationships that could be construed as a potential conflict of interest. The reviewer JT declared a shared affiliation, with no collaboration, with one of the authors, YG, to the handling Editor at the time of review.
